# Elicitation of integrated immunity in mice by a novel pneumococcal polysaccharide vaccine conjugated with HBV surface antigen

**DOI:** 10.1038/s41598-020-62185-7

**Published:** 2020-04-14

**Authors:** Wen Qian, Zhen Huang, Yuqiu Chen, Jinling Yang, Lili Wang, Kai Wu, Min Chen, Nanping Chen, Yongzhong Duan, Jing Shi, Ying Zhang, Qihan Li

**Affiliations:** 1 Institute of Medical Biology, Chinese Academy of Medicine Science & Peking Union Medical College, Yunnan Key Laboratory of Vaccine Research and Development on Severe Infectious Diseases, Kunming, 650118 China; 2 Walvax Biotechnology Co., Ltd, Kunming, 650106 China; 3 Science and Technology Achievement Incubation Center, Kunming Medical University, Kunming, 650500 China

**Keywords:** Conjugate vaccines, Drug development

## Abstract

The conjugation of polysaccharides with an effective carrier protein is critical for the development of effective bacterial polysaccharide vaccines. Therefore, the identification and optimization of carrier proteins to induce an effective immune response is necessary for developing a combined vaccine. In the current study, we utilized hepatitis B virus surface antigen (HBsAg) as a novel carrier protein combined with a capsular polysaccharide molecule to develop a new pneumococcal conjugated vaccine. The specific antibodies and T cell immune response against the capsular polysaccharide and HBsAg in the mice immunized with this conjugated vaccine were evaluated. In addition, the unique gene profiles of immune cells induced by this conjugated vaccine in the immunized mice were analyzed. Our results demonstrated that the vaccine consisting of pneumonia type 33 F capsular polysaccharide (Pn33Fps) conjugated with HBsAg can induce strong specific immune responses against both antigens *in vivo* in immunized mice. Furthermore, the conjugated vaccine induced higher expression of genes related to the activation of immunity and higher antibody titers against Pn33Fps and HBsAg in mice than those obtained via vaccination with a single antigen. Analyses of the dynamic expression changes in immunity-related genes in mice immunized with Pn33Fps_HBs, Pn33Fps, or HBsAg indicated the potent immunogenicity of the conjugated vaccine. In addition, a pathological evaluation of the organs from immunized mice further suggested that the conjugated vaccine is safe. Together, these results indicate that a conjugated vaccine consisting of Pn33Fps with HBsAg is a novel and effective vaccine.

## Introduction

Bacterial pneumonia induced by various serotypes of *Streptococcus pneumoniae* is an infectious disease with a global epidemical distribution^1^ that can to severe clinical outcomes in children, elderly adults^2^, and other age groups^3^, presenting as either invasive or noninvasive infections, including not only pneumonia but also meningitis, lethal bacteremia, otitis media and sinusitis^4,5^. This disease is an important public health issue, and the treatment and prevention of this disease are a focus worldwide^6^. Since the first pneumococcal polysaccharide vaccine was licensed in the 1980s^7^, various multivalent polysaccharide vaccines and polysaccharide-conjugated vaccines have been developed and administered to multiple populations^8,9^. These studies clarified that pneumococcal polysaccharide, as a type of T cell-independent antigen, does not directly activate T cell responses via a typical antigenic stimulating route^10,11^, which indicates that immunization with this unitary polysaccharide induces a weaker antibody response and immune memory in humans or animals^10,12^. However, the conjugation of the pneumococcal polysaccharide to a carrier protein substantially improves the specific immune response against this polysaccharide^13,14^, which results in an enhanced antibody response and an explicit memory response^15,16^. The data from a clinical trial of a recently marketed 13-valent pneumococcal conjugated vaccine further confirmed that the conjugation of pneumococcal polysaccharide molecules to carrier proteins is an effective method of inducing markedly stronger immunogenicity than that elicited by polysaccharide-alone vaccines and might represent a technical advancement in not only multivalent pneumococcal vaccines but also other bacterial vaccines^17,18^. All data obtained from these studies suggest that the conjugation of polysaccharides and carrier proteins is critical for the development of an effective bacterial polysaccharide vaccine^19^ and suggest that available carrier proteins of tetanus toxoid (TT), diphtheria toxoid (DT) and CRM197 that are widely used in other bacterial conjugate vaccines^20–22^ might lead to carrier-induced epitopic suppression (CIES)^7,23^. The impact of specific antibodies to these carrier proteins in individuals who were previously immunized with other vaccines is unclear, but this information is important for evaluating the immunization elicited by a polysaccharide-conjugated vaccine. However, the investigation of a new protein as a carrier protein is significant. Because the HBsAg vaccine has been successfully applied in the Expanded Program of Immunization (EPI) and exhibits good clinical protective effectiveness and safety in children^24,25^, the study described here investigated the hypothesis that hepatitis B surface antigen (HBsAg) might be a better carrier protein than other candidates for the development of pneumococcal conjugated vaccines. This hypothesized technical strategy leads to the design of a combined vaccine for the control of hepatitis B and pneumonia in the EPI because it could function with a new pneumococcal conjugated vaccine. Our work using the capsular polysaccharide molecule from the variant 33 F (Pn33Fps) of *Streptococcus pneumonia* type 33 A produced a conjugated vaccine according to a formulated protocol^26^. We further investigated the dynamic immune response elicited in mice inoculated with this conjugated vaccine through the detection of specific antibodies against this capsular polysaccharide and HBsAg, and the results showed a specific T cell response against both antigens. To identify the characteristic immunity and immunogenicity of this conjugated vaccine, the variation in the mRNA profile in the immune cells of immunized mice was analyzed. The data obtained in this work support the technical strategy of using pneumococcal polysaccharide-conjugated vaccines depending on the HBsAg vaccine carrier protein.

## Materials and Methods

### Hepatitis B surface antigen (HBsAg)

Hansen yeast cells containing an HBsAg antigen expression plasmid (pRMHP1.0; Technology Center of Walvax, Kunming, China) were cultured in yeast nitrogen base (YNB; Difco, USA) medium at 37 °C for 24 hours, transferred to fermentation medium (containing 150 g of glycerol, 0.5 g of NaCl, 0.5 mg of CaCl_2_, 5 g of KH_2_PO_4_, 8 g of NH_4_H_2_PO_4_, 5 g of MgSO_4_, and 2 g of KCl per liter), and cultured in this medium at 37 °C for 40 hours. Henson’s yeast cells with pRMHP1.0 were then induced to express HBsAg protein by methanol (1%, v/v) at 30 °C for 40 hours. The cells were collected by centrifugation (8000 rpm, 15 minutes), and the deposited cells were suspended for ultrasonication (2–8 °C, 5 minutes). The HBsAg protein was purified by ion-exchange chromatography (Q-Sepharose FF; GE, USA) and gel filtration (Sepharose 4FF; GE, USA)^27^.

### Pneumonia type 33 F capsular polysaccharide (Pn33Fps)

The *Streptococcus pneumoniae* 33F strain (CMCC, National Center for Medical Culture Collection, Beijing, China) was cultured in tryptic soy broth (TSB; containing 15 g of tryptic soy broth, 5 g of soybean peptone, and 5 g of NaCl per liter) medium at 37 °C for 24 hours. The supernatant containing capsular polysaccharide was harvested through centrifugal fermentation (8000 rpm, 15 minutes). The polysaccharide was purified by 20% (V/V) ethanol precipitation (30 minutes, 2–8 °C) followed by ultrafiltration (50 KD, 30 minutes, 2–8 °C) to remove bacterial nucleic acids and proteins. Finally, capsular polysaccharides (Pn33Fps) were purified by Sepharose 4FF (GE, USA)^28^.

### Conjugated vaccine (Pn33Fps_HBs)

Pn33Fps were activated by incubation with cyanogen bromide (5 mg/mL; Fluke, USA) at 2–8 °C for 20 minutes. Adipic hydrazide solution (pH 8.0 ± 0.5; Sigma, USA) was then added to an equal volume of the abovementioned solution and incubated at 2–8 °C for 30 minutes. The residual cyanogen bromide was removed by ultrafiltration (0.25 MPa, 30 minutes) to obtain the polysaccharide derivatives, and the resulting polysaccharide derivatives were mixed with HBsAg at 1:0.5 (V:V). DEPC-carbodiimide (EDAC) was then added, and the mixture was incubated at 2–8 °C for 2–4 hours. This solution was purified by column chromatography to obtain a conjugated solution (Pn33Fps_HBs).

### Ethics

The experiments using mice (Charles River Laboratories Co., Ltd., Beijing, China) were designed based on the principles established in the “Guide for the Care and Use of Laboratory Animals”^29^ and “The Guidance to Experimental Animal Welfare and Ethical Treatment”^30^. The protocol was approved by the Experimental Animal Ethics Committee of Walvax Biotechnology Co., Ltd. (approval number: Y201712001).

### Experimental design

**Figure 1:**
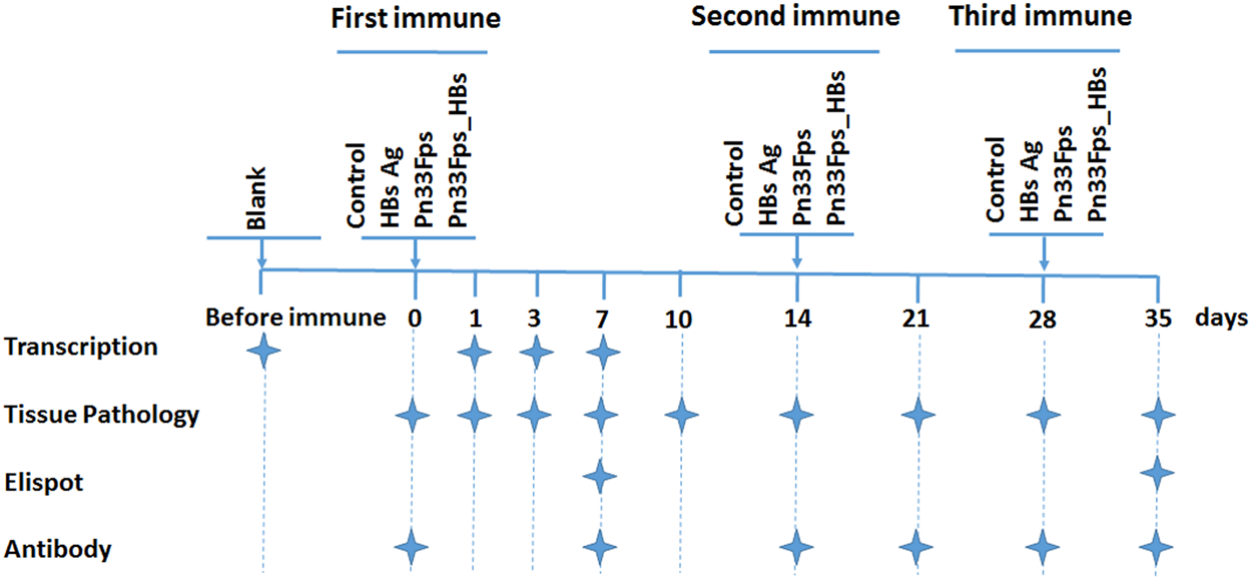
Experimental Design.

Four-week-old BALB/c female mice were divided into four experimental groups of 62 mice each: A, B, C and D. The mice in groups A, B, C and D were immunized with PBS, HBsAg (4 μg/0.2 mL per mouse), Pn33Fps (1 μg/0.2 mL per mouse), and the Pn33Fps_HBs conjugated vaccine (0.2 mL per mouse, containing 1 μg of Pn33Fps and 4 μg of HBsAg), respectively. All mice were immunized with the complex, which contains no adjuvant, via subcutaneous injection at 0, 14 and 28 days (Fig. 1). Blood samples obtained on days 0, 7, 14, 21, 28 and 35 were used for neutralization assays. Spleen samples collected on days 1, 3 and 7 were used for transcriptome assays, and those collected on days 7 and 35 were used for ELISpot assays. Tissue samples obtained on days 0, 1, 3, 7, 10, 14, 21, 28 and 35 were used for pathology analysis.

### Anti-HBsAg antibody detection

This analysis was performed using a Hepatitis B Virus Surface Antibody Detection Kit (Shanghai Kehua Bioengineering Co., Ltd.). Briefly, serum was first diluted 40-fold and then subjected to serial twofold dilutions (1:80–1:5120) in a dilution solution (0.01 M PBS; Shanghai Kehua Bioengineering Co., Ltd. China), and 50 μL of each solution was added to each well in a microtiter plate. Subsequently, 50 μL of HBsAg-HRP was added to each well (1:1000; Shanghai Kehua Bioengineering Co., Ltd. China), and the plate was then mixed well and incubated at 37 °C for 30 minutes. A developing solution (containing 0.026% TMB, 2.43 mM sodium citrate, 5.14 mM Na_2_HPO_4_, and 0.05% H_2_O_2_; Shanghai Kehua Bioengineering Co., Ltd., China) was then added, and the mixture was incubated at 37 °C for 30 minutes. Optical density (OD) values were obtained using a microplate reader (Multiskan FC; Thermo Scientific, MA, USA) at 450 nm. According to the manufacturer’s instructions, a sample with an OD value at least 2.1-fold higher than the mean OD value of the negative control serum was defined as antibody positive, whereas a sample with an OD value less than 2.1 times the mean OD value of the negative control serum was regarded as antibody negative. The positive conversion rate was calculated by dividing the number of antibody-positive mice by the total number of mice in the same group. The endpoint titers (ETs) were defined as the highest serum dilution showing a positive OD value compared with the negative control^31^. The geometric mean endpoint titer (GMET) was calculated as the geometric mean of the ETs of positive serum in the same group.

### Anti-polysaccharide antibody detection

The serum was diluted 40-fold with 0.01 M PBS (Sinopharm Chemical Reagent Co., Ltd., China) containing pneumococcal cell wall polysaccharide (CWPS, 20 μg/mL; Statens Serum Institute, Denmark) or both CWPS (20 μg/mL) and Pn22F (22F polysaccharide, 10 μg/mL; ATCC, USA) to reduce non-serotype-specific antibodies. The samples were incubated at 37 °C for 60 minutes and then at 2–8 °C overnight. A 96-well plate was coated with Pn33Fps antigen (100 ng/well) overnight at 2–8 °C. After washing with buffer (0.01 M PBS containing 0.05% Tween-20), 100 μL of a blocking solution (0.01 M PBS containing 5% skim milk powder; BD Biosciences, CA, USA) was added to each well. The place was then incubated at 37 °C for 60 minutes and washed with washing buffer. Serum was diluted 40-fold and then subjected to serial twofold dilutions (1:80–1:5120) in a dilution solution (0.01 M PBS; Sinopharm Chemical Reagent Co., Ltd., China). Serially diluted serum (100 μL/well) was added to each well, and the plate was incubated at 37 °C for 90 minutes and washed. Subsequently, 100 μL of the secondary antibody (anti-mouse IgG (H + L) (Fab)-HRP, 1:1000; Sigma, USA) was added, and the plate was incubated at 37 °C for 60 minutes. The developing solution (Sinopharm Chemical Reagent Co., Ltd. China) was then added, and the plate was incubated at 37 °C for 30 minutes. The OD values were read using a microplate reader (Multiskan FC; Thermo Scientific, MA, USA) at 450 nm. The antibody serum samples with an OD value at least 3-fold higher than the negative control value were defined as positive^32,33^. The positive conversion rate was calculated as described above. The antibody titers of serially diluted serum samples were obtained by ELISA, and the values were the highest dilutions that were considered positive^34^. The GMET was calculated based on the antibody titers of all positive serum samples in the same group.

### ELISpot assay

Mouse splenic mononuclear cells were isolated using a Lymphoprep Kit (Solarbio, Beijing, China). Mouse IFN-γ ELISpot^PLUS^ Kits and Mouse IL-4 ELISpot^PLUS^ Kits (Mabtech Inc., Nacka Strand, Sweden) were used in accordance with the manufacturer’s recommended protocol. IFN-γ- and IL-4-secreting cells stimulated with the purified HBs (s28–39) polypeptide (IPQSLDSWWTSL, 2 μg/0.1 mL per well; Sangon Biotech Co., Ltd., Shanghai, China)^35^ or with the purified Pn33Fps (5 μg/0.1 mL per well) were imaged and counted using a C.T.L. Immunospot reader (Cellular Technology Limited, OH, USA).

### Transcriptome assay

Total RNA from the spleen was extracted using an RNeasy Mini Kit (QIAGEN, GmBH, Germany) according to the manufacturer’s instructions. Transcriptome assays were conducted at BGI Genomics Co., Ltd., China, according to the procedures described in the technical manual. Briefly, total RNA was qualified and quantified using a NanoDrop and an Agilent 2100 bioanalyzer (Thermo Fisher Scientific, MA, USA). DNase I was used for the digestion of double- and single-stranded DNA in the total RNA, and magnetic beads were then used to purify and recover the reaction products. RNase H (mouse) (Illumina, USA) was used to remove the rRNA. The above-described double-stranded PCR products were heated, denatured and circularized by the splint oligo sequence. Single-stranded circular DNA (ssCir DNA) was formatted as the final library. The final library was amplified with phi29 (Thermo Fisher Scientific, MA, USA) to make DNA nanoballs (DNBs), which each contained more than 300 copies of one molecule. DNBs were loaded into a patterned nanoarray, and single-end 50-base reads were generated using the BGISEQ500 platform (BGI-Shenzhen, China). The reads were filtered using software to remove those with a low base call quality (SOAPnuke and Trimmomatic, BGI, China) and were aligned to the mouse genome (GRCm38.p5_NCBI_20180201) using Bowtie2^36,37^. The gene expression level of each sample was then calculated with RSEM^38^. Significant genes were detected according to the sequencing-based method^39^. Briefly, multiple hypothesis test corrections for the P-value of the difference were performed, and the P-value was determined by controlling the false discovery rate (FDR)^40^. The fold change in gene expression between different samples was calculated based on the gene expression level (FPKM value). Significant genes were defined as genes with an FDR ≤ 0.001 and a fold change ≥2^41,42^. The raw sequence data were deposited in the Sequence Read Archive under BioProject number PRJNA531548.

### Gene ontology (GO) analysis

GO terms were determined for the differentially expressed genes using the BGI Gene Analysis System (Dr. TOM; BGI Genomics Co., Ltd., China). Enrichment analysis was performed according to the r-hypergeometric test^43^. The P-value was determined by controlling the FDR. Significantly enriched GO terms were defined as terms with a Q value ≤ 0.05^44^.

### Protein-protein interaction (PPI) network analysis

To understand the functional interactions between the differentially expressed genes associated with immune responses, a PPI network was constructed according to protein sequence homology using the web-based tool STRING (http://www.string-db.org)^45^. Network relations with scores equal to or higher than 300 were selected for plotting. The highest scores indicated the most reliable interactions.

### qRT-PCR amplification

To confirm the accuracy of the transcriptome assay data, we randomly selected various differentially expressed genes and determined their mRNA levels by qRT-PCR using GAPDH mRNA as the internal standard. RNA was extracted from the samples using TRIzol reagent (Invitrogen Tiangen Biotech, China) according to the manufacturer's instructions. Real-time RT-PCR assays were performed using a One-Step PrimeScript RT-PCR Kit (Takara, Shuzo, Japan) with an ABI ViiA 7 Real-Time PCR System (Applied Biosystems, USA). The primers were designed using Primer Express Software v2.0 (Applied Biosystems, USA) and are shown in Table 1. The RT-PCR program was as follows: 42 °C for 5 minutes, 95 °C for 10 seconds, and 40 cycles of 95 °C for 5 seconds, followed by annealing at Tm for 30 seconds.

**Table 1:** Primers for qRT-PCR.

Gene name	Sequence (5′-3′)
Isg15	F: TCTTACCCTTTCCAGTCTGG
	R: CTCATAGATGTTGCTGTGGC
Nusap1	F: GCCACACAAAGGAAAGCTGA
	R: CCATCGTTCTTCCCTGGTTT
Ccna2	F: CTGGTCCTTCATGGAAAGCA
	R: CAGCTGCATTAAAAGCCAGG
Cd24a	F: TACCCACGCAGATTTACTGC
	R: TGGTGGTAGCGTTACTTGGA
Camp	F: TTCAACCAGCAGTCCCTAGA
	R: TTCACTCGGAACCTCACAGA
Gata1	F: TATGGCAAGACGGCACTCTA
	R: TGTTGTTGCTCTTCCCTTCC
Hmgb3	F: CAAATGCCCCCAAAAGACCT
	R: TCACCCAGCTTTTTTGCCAC
Ltf	F: ATTTCTTGAGGCCCTTGGAC
	R: TCATCTCGTTCTGCCACCTT
Aurka	F: CAACGCAAGCCAAAGGCTAA
	R: TTGCTGGTTGGCTCTTTGCT
Tal1	F: CCTGGCCAAGTTACTCAATG
	R: GGAAAGCACGTCCTGTAGAA
Tfrc	F: CCTGGCTTTCCTTCTTTCAA
	R: CAGGACAGCTTCCTTCCATT
Oas1a	F: CAGACAGCTCAGAAAAGCCA
	R: TAGCCACACATCAGCCTCTT
Abcg2	F: TTTATCCGTGGCATCTCTGG
	R: AGCATTCGCTGTGCTTGAGT
Irf7	F: AAGACCCTGATCCTGGTGAA
	R: TAGACAAGCACAAGCCGAGA
Rsad2	F: GCCGTGGTCAAGGAAAAAAG
	R: CGTCCACGTTGAAGCGATTA
GAPDH	F: GGCAAATTCAACGGCACAGT
	R: ACGACATACTCAGCACCGGC

### Histopathological examination

Tissue samples were fixed in 10% formalin, dehydrated through ethanol gradients, and embedded in paraffin, and 4-μm sections were then prepared for hematoxylin and eosin (HE) staining. The slides were visualized using a light microscope (Nikon, Tokyo, Japan).

Parameters for histopathological injury in the lung, spleen, liver and kidney were scored semiquantitatively (Table 2). During the scoring process, the pathologists were unaware of the identity of the sections they were assessing. The final score was calculated from the average of three mice per group at one time point.

**Table 2:** Scoring scheme.

Severity	Description	Score
None	No abnormality	0
Slight	Less than 15% injury	1
Mild	15%- 25% injury	2
Moderate	25%- 50% injury	3
Marked	50%-75% injury	4
Highly marked	More than 75% injury	5

### Statistical analysis

The data are shown as the means and standard deviations. Individual analyses were performed in triplicate. GraphPad Prism software (San Diego, CA, USA) was used for the statistical analyses. Student’s t tests were used when appropriate. Significant differences were defined at p < 0.05, whereas p < 0.01 indicated extremely significant differences.

## Results

### The conjugation of Pn33Fps and HBsAg can promote the immunogenicity of both antigens

**Figure 2:**
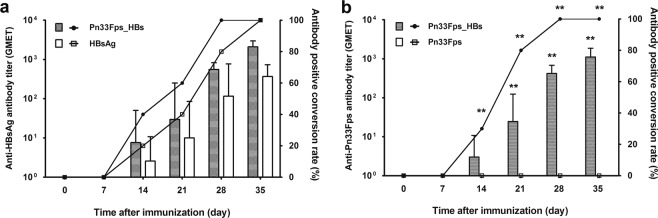
Antibodies against HBsAg (**a**) or Pn33Fps (**b**) were induced by vaccination. The antibody titer is depicted as columns, and the antibody-positive conversion rate (%) is shown as a line.

The immunogenicity of the experimental vaccine Pn33Fps_HBs in mice was analyzed through three immunizations administered at two-week intervals (Fig. 1), whereas Pn33Fps and HBsAg were administered as single antigen controls using the same immunization schedule (Fig. 1). Compared with the serum samples collected at different time points from mice immunized with the carrier protein HBsAg (Fig. 1), serum collected from mice immunized with Pn33Fps_HBs showed higher GEMT titers of antibodies specific for HBsAg, whereas both groups exhibited similar seroconversion (Fig. 2a). The highest titer of antibody against HBsAg reached 1:2099 on day 7 after the 3^rd^ inoculation in mice immunized with Pn33Fps_HBs, and this value was higher than that obtained in mice immunized with only HBsAg (1:367.7, Fig. 2a). After treatment with CWPS, the antibody against Pn33Fps in mice immunized with Pn33Fps_HBs showed a seroconversion of 100% post-2^nd^ inoculation, and the GEMT reached 1:1114 at day 7 post-3^rd^ inoculation, whereas the negative seroconversion persisted in mice inoculated with Pn33Fps alone throughout the 35-day experimental period (Fig. 2b). These observations suggest that the conjugation of Pn33Fps and HBsAg is critical for promoting the immunogenicity of both antigens, at least in mice.

### The conjugation of Pn33Fps and HBsAg can promote a specific T cell response against both antigens

**Figure 3:**
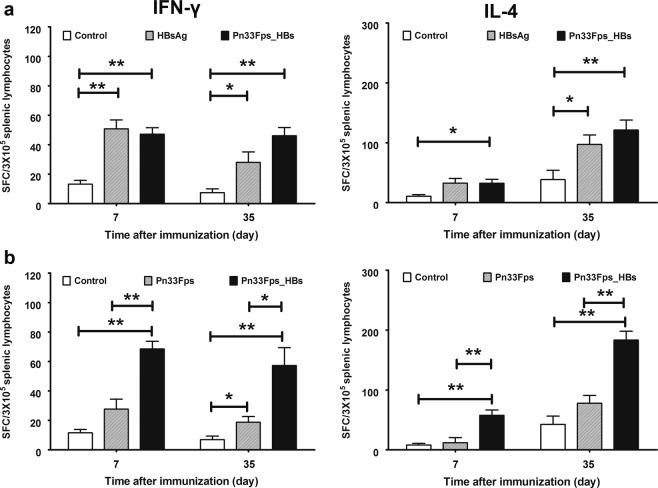
Specific T cell responses were induced by vaccination. IFN-γ- or IL-4-secreting cells stimulated with the purified HBs (s28–39) polypeptide (**a**) or purified Pn33Fps (**b**) were imaged and counted. The samples were obtained within 7 days after the first and booster immunizations. *p < 0.05, **p < 0.01.

Although previous immunological evaluations of the effectiveness of pneumococcal polysaccharide vaccines or HBsAg vaccines usually used increasing specific antibody titers as indicators^46^, ELISpot assays based on specificity for IFN-γ or IL-4 provide improved data for this immunological evaluation^47–49^. In this study, we performed ELISpot detection of specificity for IFN-γ or IL-4 in spleen cells collected from mice immunized separately with Pn33Fps, HBsAg and Pn33Fps_HBs 7 days post-1^st^ and post-3^rd^ inoculation using the peptide composed of amino acids 28–39 of HBsAg and the capsular polysaccharide of Pn33F (Pn33Fps) (Fig. 3). The results from these ELISpot detections for specificity for IFN-γ or IL-4 indicate that the specific T cell responses against HBsAg showed an obvious increase after stimulation with the synthesized peptide in cultured spleen cells collected from mice immunized with HBsAg or Pn33Fps_HBs at day 7 post-3^rd^ inoculation (Fig. 3a). Interestingly, the IFN-γ- or IL-4-specific T cell responses to the polysaccharide antigen showed a clearly higher number of spots in samples from mice immunized with Pn33Fps_HBs than in spleen cells collected from mice immunized with Pn33Fps alone (Fig. 3b). These observations suggest that the use of Pn33Fps in conjunction with the HBsAg vaccine increases the intensity of the specific T cell response.

### Characteristics of the interaction of the Pn33Fps_HBs conjugated vaccine with the immune system of mice

**Figure 4:**
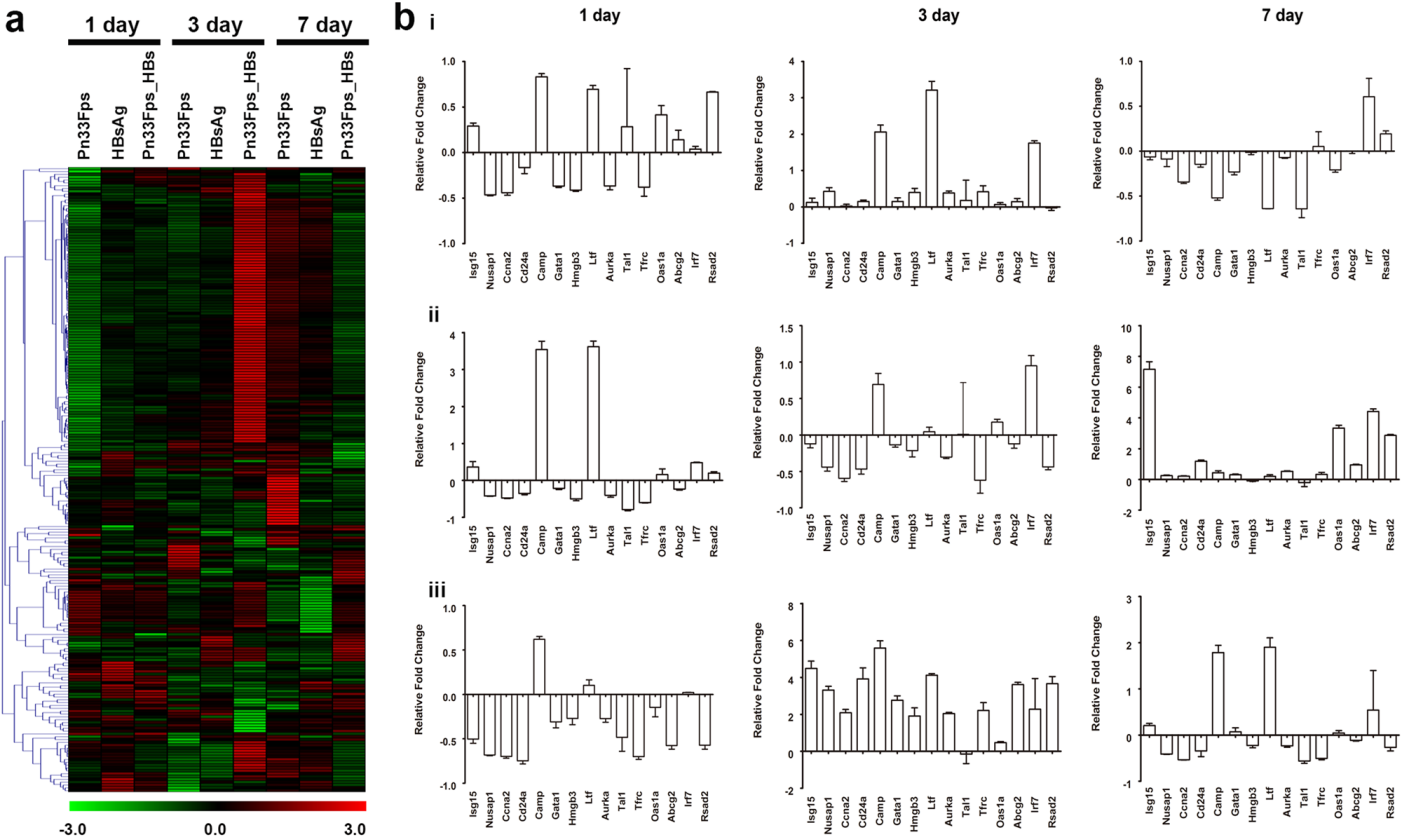
Significantly differential gene expression induced by vaccination. (**a**) Heat map and supervised hierarchical clustering analysis. Each row represents a gene, and the samples are depicted in the columns. The red color indicates the genes that were expressed at higher levels in the samples than at 0 days postinoculation (dpi), and the green color shows the genes that were expressed at lower levels in the samples than at 0 dpi. The colored bars represent log2 of the fold change. (**b**) Confirmation of gene expression changes by qRT-PCR. A total of 15 individual genes in the HBsAg (i), Pn33Fps (ii) and Pn33Fps_HBs (iii) groups were randomly selected and analyzed by qRT-PCR. The results are normalized to the GAPDH expression level. The individual analyses were performed in triplicate. The error bars indicate the means ± SDs.

Current immunological knowledge indicates that the phenotype of the immune response elicited by a vaccine antigen is determined based on the characteristics of the interaction of the antigen with the immune system^50^, which includes the recognition of the antigen structure by innate immunity when the vaccine is inoculated into epithelial or muscular tissue^51^ and the presentation of the antigen peptide to T cells by dendritic cells, macrophages and innate lymphoid cells^13,51^. This process is dynamically involved in the variation in mRNA transcript expression in various immune cells^52^ and might serve an indicator of the characteristics of the interaction between the vaccine antigen and the immune system^53,54^. Here, we collected spleen cells from mice immunized with Pn33Fps, HBsAg and Pn33Fps_HBs at days 1, 3, and 7 post-1^st^ inoculation (Fig. 1) and compared their mRNA gene expression profiles. The overall results showed that the mRNA levels of genes related to immune response were clearly higher in mice immunized with Pn33Fps_HBs at day 3 than in mice immunized with a single antigen. Interestingly, most of these genes were downregulated at day 7 in the mice immunized with Pn33Fps_HBs and were upregulated in the mice immunized with Pn33Fps or HBsAg (Fig. 4a). This result suggests a characteristic interaction of Pn33Fps_HBs with the immune system over time, which indicates that Pn33Fps_HBs, as a conjugated antigen complex, might stimulate the innate immune system early, whereas this stimulation might occur later during immunization with a single antigen, such as Pn33Fps and HBsAg. To further confirm the variation in mRNA gene expression obtained in the above microarray analysis, 15 up- or downregulated genes were randomly selected for verification by qRT-PCR, and the results supported the data described above (Fig. 4b).

### Characterization of gene expression in mice immunized with Pn33Fps_HBs, Pn33Fps and HBsAg

**Figure 5:**
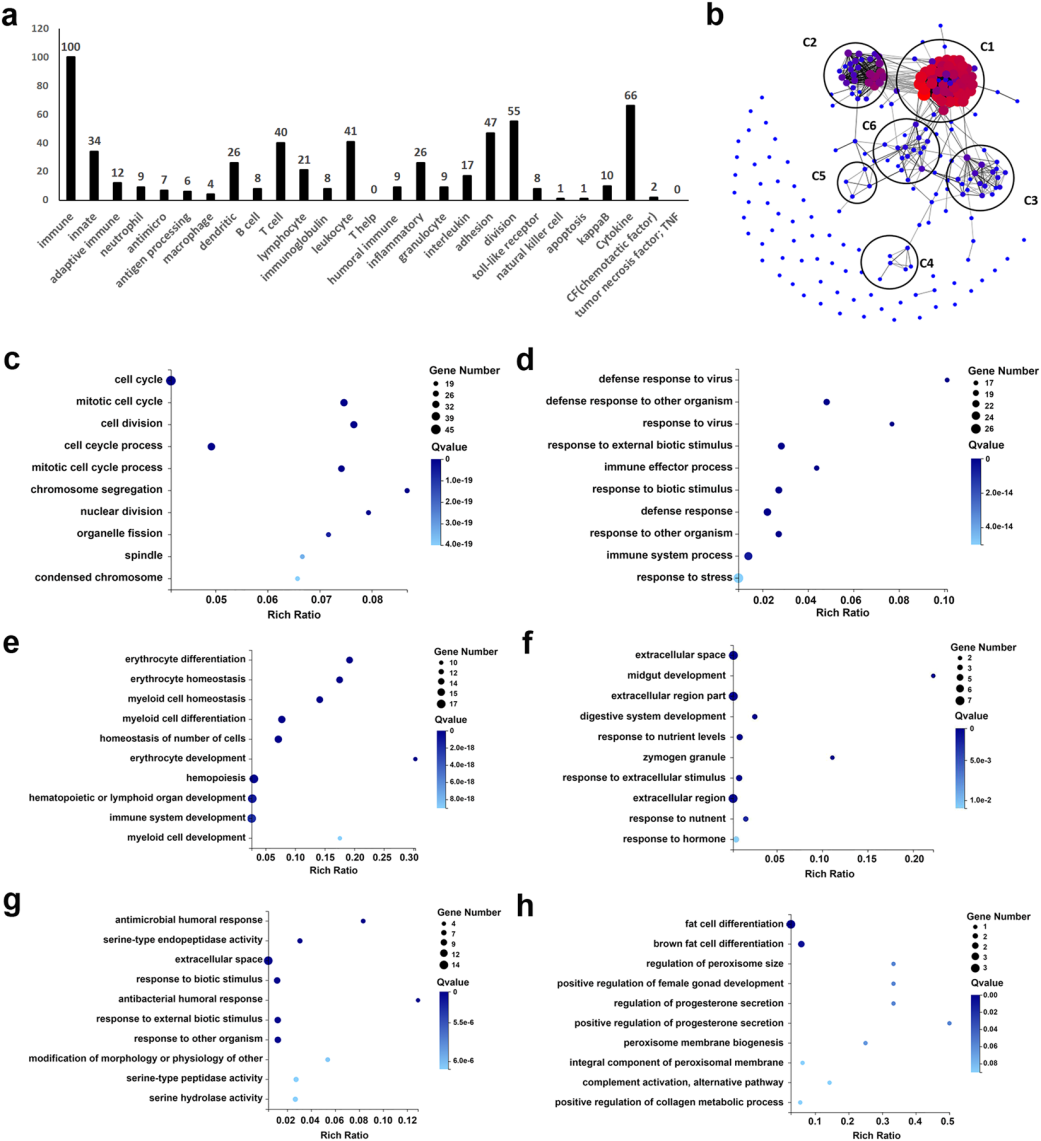
Gene expression changes were induced by the vaccine in mice. (**a**) GO analysis of the 651 differentially expressed genes in spleen cells collected 0, 1, 3 and 7 dpi that were associated with immune responses. (**b**) Cluster analysis of the 220 differentially expressed genes associated with immune responses according to protein sequence homology. (**c**) GO enrichment analysis of the differentially expressed genes belonging to the first cluster (C1). (**d**) GO enrichment analysis of the differentially expressed genes belonging to the second cluster (C2). (**e**) GO enrichment analysis of the differentially expressed genes belonging to the third cluster (C3). (**f**) GO enrichment analysis of the differentially expressed genes belonging to the fourth cluster (C4). (**g**) GO enrichment analysis of the differentially expressed genes belonging to the fifth cluster (C5). (**h**) GO enrichment analysis of the differentially expressed genes belonging to the sixth cluster (C6).

The abovementioned data indicate that the Pn33Fps_HBs conjugated vaccine elicits a characteristic antigenic stimulation of the immune system that differs from that induced by the Pn33Fps or HBsAg vaccine. The conjugated vaccine induced not only antibody titers against both antigens (Figs. 2 and 3) but also variations in mRNA gene expression in immune cells (Fig. 4). Based on these data, we further clarified the functions of genes that were differentially expressed in the three groups and found 220 genes that are directly involved in 25 immune functions and were differentially expressed in all of the groups (Fig. 5a). A cluster analysis of these 220 genes revealed six clusters (C1-C6) through structure-function identification of the protein sequences encoded by the genes (Fig. 5b). Further GO analysis of these six clusters showed the functional distribution of the genes: the genes belonging to cluster 1 are functionally related to cellular cycle regulation (Fig. 5c), which implies the proliferation tendency of immune cells under antigen stimulation. In addition, most of the genes in C2, C3, C4 and C5 are clearly involved in the immune response (Fig. 5d–g), and the genes in C6 are related to adipocyte differentiation, which a previous study indicated is potentially involved in immunity (Fig. 5h)^55^. These results from the gene functional clarification analysis suggest that the expression profile of responsive genes in immunized mice elicited by the conjugated vaccine Pn33Fps_HBs differs from that obtained in the other two groups, and this difference reflects a characteristic immune activation elicited by Pn33Fps_HBs.

### Dynamic differences in mRNA gene expression in mice immunized with Pn33Fps_HBs, Pn33Fps and HBsAg

**Figure 6:**
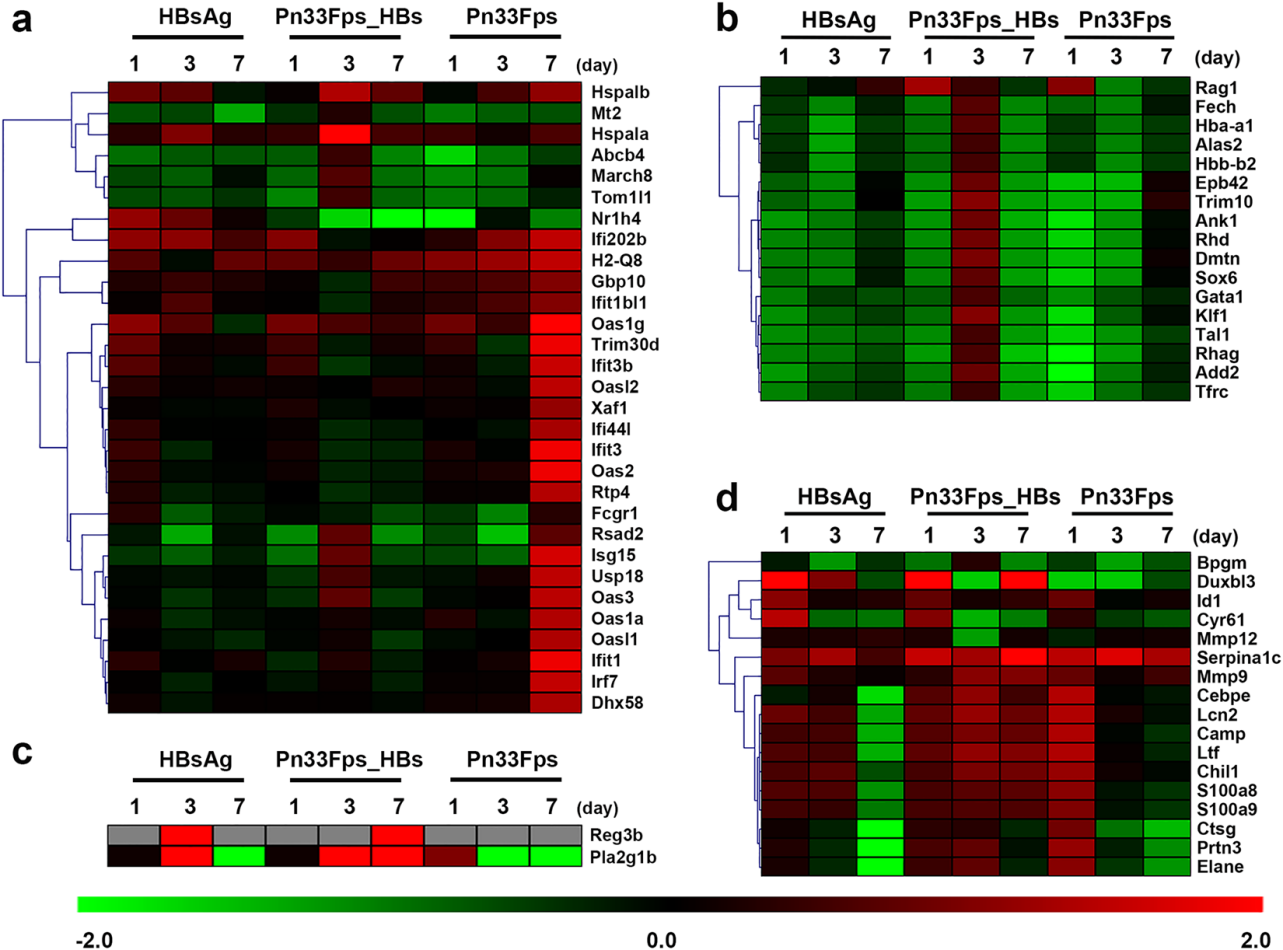
Fold changes in the expression of different genes in C2-C5 at different time points. (**a**) Fold change in the expression of different genes belonging to the second cluster (C2) at different time points. (**b**) Fold change in the expression of different genes belonging to the third cluster (C3) at different time points. (**c**) Fold change in the expression of different genes belonging to the fourth cluster (C4) at different time points. (**d**) Fold change in the expression of different genes belonging to the fifth cluster (C5) at different time points. The colored bars represent log2 of the fold change.

To investigate the characteristics of the immune response elicited by the conjugated vaccine Pn33Fps_HBs, the dynamic differences in gene expression in the Pn33Fps_HBs-immunized mice were compared with those in Pn33Fps- or HBsAg-immunized mice. The comparisons of the C2 genes show obvious differences between the Pn33Fps_HBs group and the Pn33Fps group, as indicated by the upregulation of some genes at day 7 postinoculation, but no difference from the HBsAg group (Fig. 6a). Most of the C3 genes showed clear differences between the Pn33Fps_HBs group and the Pn33Fps and HBsAg groups at day 3 postinoculation, as demonstrated by upregulation in the Pn33Fps_HBs group and downregulation in the two other groups (Fig. 6b). The dynamic expression of the C4 genes in the Pn33Fps_HBs group showed a similar trend to that in the HBsAg group but different from that in the Pn33Fps group (Fig. 6c). The expression of C5 genes was upregulated until day 7, whereas the expression of these genes in the Pn33Fps and HBsAg groups returned to normal levels (Fig. 6d). These results suggest that the immunity elicited by conjugated vaccines differs from that induced by polysaccharide molecules or peptide vaccines but also identified some genes and their encoding proteins that are most likely involved in the formation of effective immunity. Additionally, these results imply that this conjugated vaccine induces an integrated immune response with activated T cells against pneumococcal polysaccharide and HBsAg. Further investigation of these genes might provide additional data.

### Pathology observation of the organs of mice immunized with Pn33Fps_HBs, Pn33Fps or HBsAg

**Figure 7:**
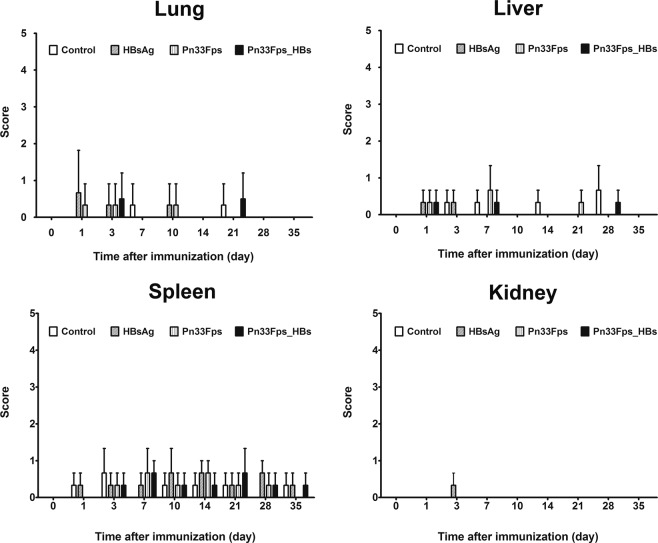
Pathological changes in the organs of mice immunized with the Pn33Fps_HBs conjugated vaccine, Pn33Fps or HBsAg antigen. Histopathological injury was scored semiquantitatively using the scoring standards shown in Table 2.

The goal of the development of a conjugated vaccine (Pn33Fps_HBs) is to improve the immunogenicity of pneumococcal polysaccharides because the HBsAg vaccine plays a role in immunization in children. For a conjugated vaccine with the characteristics of a combined vaccine, safety is a concern. Here, we also performed detailed histopathology observations of various organs of mice immunized with this conjugated vaccine in comparison with those of mice immunized with Pn33Fps or HBsAg. The results suggest that various organs collected from mice immunized with the conjugated vaccine, Pn33Fps_HBs, exhibited no obvious pathological lesions compared with those of Pn33Fps- or HBsAg-immunized mice, which presented only sporadic and slight inflammatory cell aggregation in the spleen (Fig. 7).

## Discussion

During the development of bacterial vaccines, the immunogenicity of capsular polysaccharide molecules is enhanced using various technical strategies^56^. Traditionally, the conjugation of the carrier proteins TT, DT and CRM197 with bacterial polysaccharide provides a technical strategy for optimizing the immunogenicity of polysaccharide antigens and suggests the potential of this technical strategy^14,57^. However, the fact that these carrier proteins are actually the antigenic components of some EPI vaccines, such as DTP, suggests the significance of searching for innovative carrier proteins^58^. Our study investigated a new technical strategy for the development of an innovative polysaccharide-conjugated vaccine with an HBsAg vaccine as the carrier protein, which suggests the design of a combined vaccine composed of bacterial and viral antigens and identifies a new carrier protein. Undoubtedly, there are many technical and theoretical concerns regarding this strategy. However, the study of one polysaccharide molecule, Pn33Fps, conjugated to an HBsAg peptide highlights the possibility of developing a multivalent pneumococcus polysaccharide conjugated to this carrier protein in the future.

Previous studies have indicated that pneumococcal capsular polysaccharide antigen elicits an immune response in humans^59^, and its immunogenicity is enhanced by conjugation to a carrier protein^60^. However, the capsular polysaccharide is usually unable to elicit an antibody response in mice^61^, which implies that stimulation with this polysaccharide antigen fails to reach a threshold value of effective immunity in mice^62^. Our results demonstrate a high specific antibody response and a T cell response, which suggest the availability of a mouse model for the immunological study of polysaccharide-conjugated vaccines with HBsAg as the carrier protein because this model can be used for the evaluation of an HBsAg vaccine^63^. Our observations indicate that the Pn33Fps_HBs conjugated vaccine not only elicits antibody against the Pn33Fps antigen at a high level but also elicits a higher titer of antibody against the HBsAg antigen, and both effects are increased by boost immunizations. However, the same quantity of polysaccharide antigen did not induce a positive conversion rate or antibody titer even if the immunization was boosted. Antibodies against HBsAg were elicited by the conjugate vaccine earlier than by HBsAg alone and reached a tier up to 2099 higher. These results suggest that the comprehensive immunogenicity of this conjugated vaccine enables the elicitation of an enhanced immune response against both antigens, which is similar to previously reported results^7,46,64^. The finding that T cell responses specific to IFN-r and IL-4 were elicited suggests that this conjugated vaccine raised an integrated immunity in immunized mice. Importantly, our findings suggest that the HBV peptide vaccine, as a carrier protein for pneumococcal polysaccharides, not only enhances the immunogenicity of polysaccharides but also shows a possible technical method for new combined vaccines because the HBV and pneumococcal vaccines are administered with similar immune schedules^65,66^. Furthermore, the dynamic comparisons of the immunological mRNA profile of the Pn33Fps_HBs group with those of the Pn33Fps and HBsAg groups suggest that the stronger immunity elicited by this conjugated vaccine compared with that elicited by each individual antigen is likely due to different antigenic stimulation of the immune system and to the characteristic expression of some immune signal molecules in various innate and adaptive immune cells, which leads to discrepant immune responses. The various modulated genes in these processes probably imply the mechanism involved in these discrepant immunities, and studies on the expression of these genes would improve the technical design of a multivalent pneumococcus polysaccharide-conjugated vaccine using HBsAg protein. Our work thus identifies not only the immunological efficacy of this monovalent Pn33Fps_HBs conjugated vaccine but also its safety through pathological observation.

Based on the epidemic status of pneumonia and hepatitis B in the pediatric population^67,68^, our data provide a technical route for the development of new combined vaccines against various diseases, including hepatitis B and pneumonia. Although many points regarding bacterial conjugated vaccines obtained using this new strategy should be considered, the notion of using a viral peptide vaccine as a carrier protein is significant for the development of a new generation of combined vaccines.

## Supplementary information

Supplemental Figure 1.
