# Supplementary material for: Elicitation of integrated immunity in mice by a novel pneumococcal polysaccharide vaccine conjugated with HBV surface antigen
Source: Sci Rep. 2020 Apr 14;10:6470. doi: 10.1038/s41598-020-62185-7 (PMC7156719; doi:10.1038/s41598-020-62185-7)
Supplement: Supplementary file 1 — Supplemental Figure 1. [file 41598_2020_62185_MOESM1_ESM.docx]

*Supplementary information*

**Elicitation of integrated immunity in mice by a novel pneumococcal polysaccharide vaccine conjugated with HBV surface antigen**

Wen Qian^1,2^, Zhen Huang^2^, Yuqiu Chen^2^, Jinling Yang^1^, Lili Wang^2^, Kai Wu^2^, Min Chen^2^, Nanping Chen^2^, Yongzhong Duan^1,3^, Jing Shi^2^, Ying Zhang^1,*^, Qihan Li^1,*^

1, Institute of Medical Biology, Chinese Academy of Medicine Science & Peking Union Medical College, Yunnan Key Laboratory of Vaccine Research and Development on Severe Infectious Diseases, Kunming 650118, China

2, Walvax Biotechnology Co., Ltd., Kunming 650106, China

3, Science and Technology Achievement Incubation Center, Kunming Medical University, Kunming 650500, China


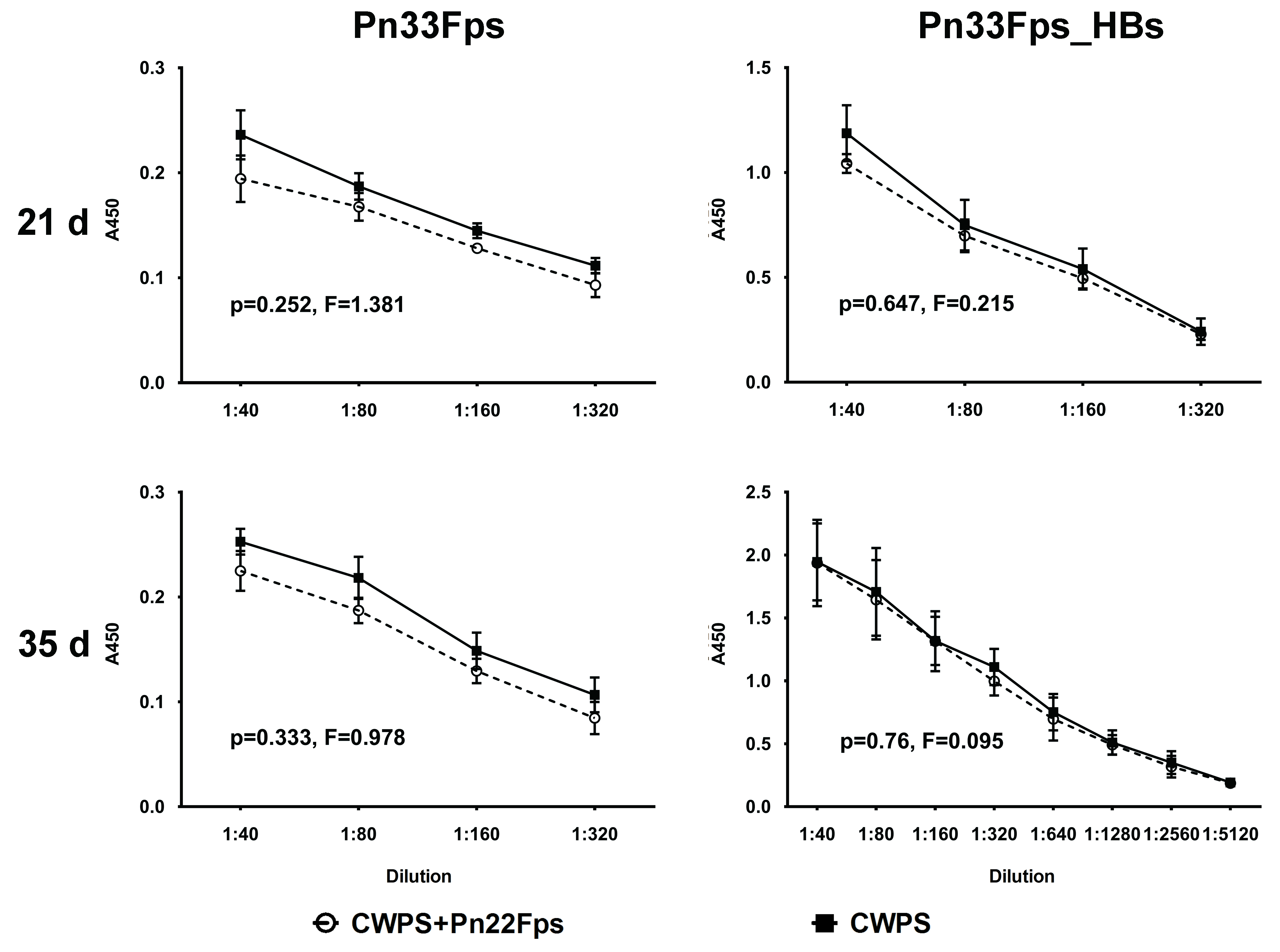


**Supplemental Figure 1. Comparison of the differences in the effects of CWPS and CWPS+Pn22F pre-absorption on antibody titer detection.**

The differences between the two pre-absorption methods were analyzed by repeated measures analysis of variance (ANOVA). The X-axis showed the serum dilution, and the Y-axis indicates the light absorbance value at a wavelength of 450 nm. Mean ± 95% CI.
